# Advances in understanding the molecular basis of skin fragility

**DOI:** 10.12688/f1000research.12658.1

**Published:** 2018-03-06

**Authors:** Cristina Has

**Affiliations:** 1 Department of Dermatology and Venerology, Medical Center – University of Freiburg, Faculty of Medicine, University of Freiburg, Hauptstrasse 7, DE-79104, Freiburg, Germany

**Keywords:** Skin fragility, epidermolysis bullosa, genetic causes

## Abstract

Skin fragility refers to a large group of conditions in which the ability of the skin to provide protection against trivial mechanical trauma is diminished, resulting in the formation of blisters, erosions, wounds, or scars. Acquired and physiological skin fragility is common; genetic disorders are rare but give insight into the molecular mechanisms ensuring skin stability. The paradigm is represented by inherited epidermolysis bullosa. This review is focused on recent advances in understanding the molecular basis of genetic skin fragility, including emerging concepts, controversies, unanswered questions, and opinions of the author. In spite of the advanced knowledge on the genetic causes of skin fragility, the molecular pathology is still expanding. Open questions in understanding the molecular basis of genetic skin fragility are the following: what are the causes of phenotypes which remain genetically unsolved, and what are the molecular modifiers which might explain phenotypic differences among individuals with similar mutations?  New mutational mechanisms and new genes have recently been discovered and are briefly described here. Comprehensive next-generation sequencing-based genetic testing improved mutation detection and facilitated the identification of the genetic basis of unclear and new phenotypes. Characterization of the biochemical and cell biological consequences of the genetic variants is challenging and laborious but may represent the basis for personalized therapeutic approaches. Molecular modifiers of skin fragility have been uncovered in particular animal and genetic models but not in larger cohorts of patients. This scientific progress is the basis for revisions of the epidermolysis bullosa classification and for innovative therapeutic approaches designed for this intractable condition.

## Molecular basis of skin fragility

Skin fragility refers to a broad range of conditions in which the ability of the skin to provide protection against trivial mechanical stressors is not fully ensured, resulting in the formation of blisters, erosions, wounds, and ultimately scars. It is essentially due to the weakening (for example, decreased amount or functionality) of the structures which ensure cutaneous stability. The mechanical resilience of the cutaneous organ relies mainly on multimolecular suprastructures, which provide stable attachment of epidermal keratinocytes to each other (that is, desmosomes and tight junctions) and to the underlying connective tissue (that is, hemidesmosomes, focal adhesions, basement membrane, and anchoring fibrils) and regulate tissue homeostasis in critical cell processes that include tissue barrier function, cell proliferation, and migration. Furthermore, the keratin cytoskeleton confers structural support and deformability on keratinocytes, while the extracellular matrix and the collagen and elastic fibers are suited to cushion mechanical forces.

The relevance of the topic is high, since skin fragility is common and occurs in physiological, pathological, or iatrogenic situations. For example, neonatal and senescent skin is physiologically fragile because of functional immaturity or decline, respectively ^1, 2^. Acquired fragility of the skin is frequent in the clinical practice being associated with high morbidity and costs. It can be induced by photo damage, corticosteroids, diabetes, peripheral vascular disorders, autoimmune processes, and so on. Genetic disorders provide excellent models to understand the pathogenic mechanisms underlying skin fragility ^3^. The prototype of genetic skin fragility disorders is represented by inherited epidermolysis bullosa (EB), which is the focus of this review. Other groups of disorders, such as peeling skin disorders, keratinopathic ichthyoses, pachyonychia congenita, and Ehlers-Danlos syndromes, also display features of skin fragility but will not be discussed here ^3^. Mutations of the genes encoding the most prominent proteins with structural and scaffolding functions in the skin—such as keratins 5 and 14, plectin, bullous pemphigoid antigen 1e (BPAG1e), collagen XVII, integrin α6β4, laminin 332, or collagen VII—account for most cases of inherited EB. Based on the advances in understanding the molecular basis of skin fragility, the revised recommendations for EB classification extended the EB spectrum beyond the classic types to comprise disorders such as the Kindler syndrome, the acral peeling skin syndrome, the acantholytic EB, and desmosomal skin fragility diseases ^4, 5^.

Open questions in understanding the molecular basis of genetic skin fragility are (a) what are the causes of about 15% of the phenotypes which remain genetically unsolved by Sanger sequencing of candidate genes ^6^ and (b) what are the molecular modifiers which might explain phenotypic differences among individuals with similar mutations? The results of comprehensive next-generation sequencing (NGS)-based genetic testing for EB published in the last four years shed some light on these questions.

## Old and new players in the mechanical stability of the skin

Most studies using NGS-based genetic testing in EB cases in which clinical assessment, skin biopsy analysis, and/or Sanger sequencing of candidate genes had failed subclassification or identification of pathogenic mutations nevertheless revealed mutations in genes known to be associated with EB ^6– 10^. This finding is not unexpected and points to the fact that the clinical and molecular pathology of skin fragility is multifaceted and highly complex. Classic genotype-phenotype correlations in which lack of gene products, such as collagen VII or laminin 332, lead to clear-cut clinical presentations of severe generalized dystrophic or junctional EB are just the “tip of the iceberg”. At the “bottom of the iceberg”, the spectrum of genetic skin fragility extends to mild phenotypes, which may remain underdiagnosed. These are characterized by one or more of the following features: occurrence of erosions, wounding, or scarring after minimal scratches but no blister formation, localized/acral blistering, onset of clinical manifestations in childhood or adult age, or improvement of the clinical manifestations with age. Such phenotypes result from mutations which induce moderate alterations in the abundance (that is, expression, stability, and size) or functionality (that is, processing, interactions, and dimerization/trimerization) of the respective proteins (examples reported in 11, 12). On the other hand, comprehensive genetic analyses identified variants of uncertain/unknown significance (VUSs) ^13^, which were predicted to affect gene transcription or splicing. Exhaustive workup, including RNA sequencing and protein biochemistry, is required to prove the pathogenicity of such VUSs and explain genotype-phenotype correlations (examples reported in 14– 18).

Besides these general remarks, some genes/proteins and phenotypes are particularly interesting.

One example is plectin, a huge, 500 kD plakin protein of the inner plaque of the hemidesmosomes. Its complexity is due to the existence of multiple isoforms with tissue-specific expression, a multitude of interaction partners within the intermediate filament, and actin adhesion complexes (reviewed in 19– 23). Mutations in the gene encoding plectin, *PLEC*, result in “plectinopathies”, including cutaneous or extracutaneous features: EB simplex with muscular dystrophy (MIM 226670), EB simplex with pyloric atresia (MIM 612138), EB simplex Ogna (MIM 131950), and muscular dystrophy, limb-girdle, type 2Q (MIM 613723). EB simplex Ogna is the only autosomal dominant condition associated with PLEC mutations. It was first recognized as a distinct disorder and later genetically solved by the late Tobias Gedde-Dahl and colleagues ^24, 25^. Clinically, it resembles localized or generalized intermediate EB simplex and lacks muscular dystrophy. It is caused by a specific amino acid substitution, exchanging an arginine residue with a tryptophan at position 2000, p.Arg2000Trp in the rod domain of plectin ^26, 27^. The molecular pathology was elucidated in detail in the mouse model ^28^. The mutation p.Arg2000Trp renders the coiled-coil rod domain of plectin more vulnerable to cleavage by calpains and other proteases activated in the epidermis but not in skeletal muscle. This results in insufficient protein levels of the hemidesmosome-associated plectin isoform 1a, which is required for efficient hemidesmosome formation ^28^.

In 2015, a mutation in exon 1a of *PLEC* (c.46C>T, p.Arg16Ter), leading to the disruption of plectin isoform 1a, the dominant isoform in the epidermal basal cell layer, was shown to cause a new phenotype: autosomal-recessive skin-only EB simplex (designated as EB simplex with nail dystrophy, MIM 616487) ^29^. Skin disease started with foot blisters at walking age and became generalized at puberty while sparing mucous membranes ^29^. This phenotype was associated with hypoplastic hemidesmosomes, but screening for cardiomyopathy and muscle dystrophy showed no abnormalities ^29^.

A similar example is the BPAG1, encoded by the dystonin gene ( *DST*). The complexity also resides in the existence of different isoforms (epithelial, neuronal, and muscular) and molecular interactions (reviewed in 30). In humans, *DST* mutations, affecting distinct BPAG1 isoforms, lead to two autosomal-recessive disorders: (a) neuropathy, hereditary sensory and autonomic, type VI (MIM 614653) and (b) EB simplex, autosomal recessive 2 (MIM 615425). The epithelial isoform BPAG1e was considered a major scaffolding molecule in hemidesmosomes. However, mutations leading to premature termination codons in the coiled-coil domain led to a rather mild EB simplex, manifesting with lifelong trauma-induced blisters and erosions particularly affecting the extremities ^31– 33^, while distal truncation of BPAG1e was associated with EB simplex generalized intermediate with prurigo papules ^34^. Recently, the first case with a mutation affecting an exon expressed in both the neuronal and the epithelial isoforms and a complex phenotype was reported ^35^. Whole exome sequencing revealed compound heterozygous *DST* variants—c.3886A>G, p.Arg1296Ter in exon 29 (expressed in both epithelial and neuronal isoforms) and c.806C>T, p.His269Arg in exon 7 (included in the neuronal isoform)—in a 17-year-old female presenting with a complex phenotype consisting of both skin and neuronal involvement as well as iris heterochromia, cataract, hearing impairment, syringomyelia, behavioral and gastrointestinal issues, osteoporosis, and growth hormone deficiency ^35^.

Besides, the kelch-like protein 24 (KLHL24) emerged as a new contributor to the stability of the skin ^36, 37^. Although an explanation for the genetically unsolved EB simplex cases was sought for many years, identification of *KLHL24* mutations was unexpected and very little was known about this gene/protein ^38^. The kelch-like family (KLHL proteins) comprises 42 proteins which contain a BTB domain which binds to cullin 3, a scaffold protein required for ubiquitination and proteasomal degradation of substrate proteins ^39, 40^. KLHL24 is expressed in the main skin cell types—keratinocytes, fibroblasts, and melanocytes—and in other tissues such as brain, liver, heart, skeletal muscle, kidney, pancreas, placenta, lung, and peripheral blood ^36, 37^. In neurons, KLHL24 binds to the C-terminal domain of the kaianate receptor GluR6 and regulates its function by interacting with PICK1 (protein interacting with protein kinase C, alpha) ^41^. All 26 EB simplex patients reported so far harbored monoallelic mutations in the translation initiation codon of *KLHL24*, c.1A>G, c.1A>T, c.2T>C, c.3G>T, c.3G>A ^36, 37, 42^. The consequence is the usage of a transcription initiation site located 29 codons downstream, resulting in an N-terminal truncation. Based on experiments in recombinant expression systems, KLHL24 was proposed to function as a cullin 3–Rbx1 ubiquitin ligase substrate receptor for keratin 14. N-terminal truncated KLHL24 was more stable than its wild-type counterpart because of abolished autoubiquitination. This gain-of-function led to loss of keratin 14 in the skin of one patient and in a mouse model ^36^. Although this straightforward hypothesis is very attractive, neither keratin 14 nor keratin 5 was significantly reduced in the skin or keratinocytes of the patients reported by other groups ^37, 42^. New reports are expected to clarify this controversy.

## Molecular modifiers of skin fragility

Two exciting articles identifying modifiers in EB were published in 2014. Sproule *et al*. used a mouse model with a hypomorphic laminin γ2 (Lamc2) allele that recapitulates generalized junctional EB to demonstrate the potent impact of genetic modifiers on the strength of dermal-epidermal adhesion and on the clinical severity of junctional EB ^43^. Through an unbiased genetic approach involving a combination of quantitative trait locus (QTL) mapping and positional cloning, the authors demonstrated that *Col17a1* is a strong genetic modifier of the junctional EB that develops in Lamc2-deficient mice. This modifier is defined by variations in 1–3 neighboring amino acids in the non-collagenous 4 domain of the collagen XVII protein ^43^. These allelic variants alter the strength of dermal-epidermal adhesion in the context of the Lamc2 mutation and, consequentially, broadly impact the clinical severity of junctional EB ^43^.

Odorisio *et al*. used an exceptional genetic model to uncover molecular modifiers in dystrophic EB ^44^. They studied a monozygotic twin pair with recessive dystrophic EB presenting different phenotypic manifestations while expressing similar amounts of collagen VII ^44^. Gene expression analysis in the twins’ fibroblasts showed differential expression of genes associated with transforming growth factor-beta (TGF-β) pathway inhibition. Decorin, a skin matrix component with anti-fibrotic properties, was more abundant in the less affected twin, while both TGF-β canonical and non-canonical pathways were more activated in the fibroblasts of the more affected twin. These data showed that the amount of type VII collagen is not the only determinant of clinical severity and indicated an involvement of TGF-β pathways in modulating disease variability ^44^.

## Looking back and forward

- In spite of the advanced knowledge on the molecular basis of skin fragility, the molecular pathology is still expanding. New mutational mechanisms, genes, and phenotypes still emerge.
- Comprehensive NGS-based genetic testing improved mutation detection and facilitated the identification of the genetic basis of unclear phenotypes. However, this approach did not reveal any novel genetic modifiers in large cohorts of patients.
- Understanding the biochemical and cell biological consequences of the mutations remains challenging. Such studies significantly deepen our knowledge and represent the basis for personalized therapeutic approaches. They are very laborious and usually not rewarded by the journal impact factor.
- Detailed characterization of the functions of the proteins which were recently implicated in EB, exophilin 5, and KLHL24 is required. Therapeutic implications may emerge.
- EB is one of the privileged “orphan diseases” because the classification has been regularly revised in past decades according to the scientific progress. The complexity of molecular and clinical constellations has constantly increased, rendering the classification system quite complicated for non-specialists. The dilemma is to lump or to split. The aims should be to provide a pragmatic tool and achieve broad adherence and implementation in clinical practice.
- The rigorous and tenacious research of the molecular bases and pathomechanisms of EB is currently rewarded by impressive progress made in EB therapy. Cell therapies, such as bone marrow transplantation ^45^ and local or systemic injections with fibroblasts ^46^ or mesenchymal stromal cells ^47^, showed benefit in reducing wounds, inflammation, and pain in patients with dystrophic EB. Remarkably, the entire epidermis of a boy with junctional EB could be regenerated by transgenic stem cells ^48^. Aminoglycosides proved effective in providing read-through of premature termination codons in patients with dystrophic EB ^49^, while clinical trials are ongoing to demonstrate the effect of anti-TGF- β therapies. Though not available to all people with EB, these procedures provide hope for the future.

## Abbreviations

BPAG1, bullous pemphigoid antigen 1; EB, epidermolysis bullosa; KLHL24, kelch-like protein 24; Lamc2, laminin γ2; NGS, next-generation sequencing; TGF-β, transforming growth factor-beta; VUS, variant of uncertain significance.
